# Alterations of the gut virome in patients with systemic lupus erythematosus

**DOI:** 10.3389/fimmu.2022.1050895

**Published:** 2023-01-11

**Authors:** Changming Chen, Qiulong Yan, Xueming Yao, Shenghui Li, Qingbo Lv, Guangyang Wang, Qin Zhong, Fang Tang, Zhengqi Liu, Ying Huang, Yang An, Jing Zhou, Qiongyu Zhang, Aiqin Zhang, Hayan Ullah, Yue Zhang, Can Liu, Dan Zhu, Hufan Li, Wen Sun, Wukai Ma

**Affiliations:** ^1^ Department of Rheumatology and Immunology, The Second Affiliated Hospital of Guizhou University of Traditional Chinese Medicine, Guiyang, China; ^2^ Department of Microbiology, College of Basic Medical Sciences, Dalian Medical University, Dalian, China; ^3^ Puensum Genetech Institute, Wuhan, China; ^4^ College of Animal Science and Veterinary Medicine, Heilongjiang Bayi Agricultural University, Daqing, China; ^5^ Key Laboratory of Health Cultivation of the Ministry of Education, Beijing University of Chinese Medicine, Beijing, China

**Keywords:** systemic lupus erythematosus, gut virome, bulk metagenome, virus-like particle-based metagenome, viral diversity, viral dysbiosis

## Abstract

**Background:**

Systemic lupus erythematosus (SLE) is a systemic autoimmune disease that has been linked to the dysbiosis of the gut microbiome and virome. However, the potential characterization of the gut virome in SLE patients needs to be explored more extensively.

**Methods:**

Herein, we analyzed the gut viral community of 16 SLE patients and 31 healthy controls using both bulk and virus-like particle (VLP)-based metagenomic sequencing of their fecal samples. A total of 15,999 non-redundant viral operational taxonomic units (vOTUs) were identified from the metagenomic assembled contigs and used for gut virome profiling.

**Results:**

SLE patients exhibited a significant decrease in gut viral diversity in the bulk metagenome dataset, but this change was not significant in the VLP metagenome dataset. Also, considerable alterations of the overall gut virome composition and remarkable changes in the viral family compositions were observed in SLE patients compared with healthy controls, as observed in both two technologies. We identified 408 vOTUs (177 SLE-enriched and 231 control-enriched) with significantly different relative abundances between patients and controls in the bulk virome, and 18 vOTUs (17 SLE-enriched in 1 control-enriched) in the VLP virome. The SLE-enriched vOTUs included numerous *Siphoviridae*, *Microviridae*, and *crAss-like* viruses and were frequently predicted to infect *Bacteroides*, *Parabacteroides*, and *Ruminococcus_E*, while the control-enriched contained numerous members of *Siphoviridae* and *Myoviridae* and were predicted to infect *Prevotella* and *Lachnospirales_CAG-274*. We explored the correlations between gut viruses and bacteria and found that some *Lachnospirales_CAG-274* and *Hungatella_A* phages may play key roles in the virus-bacterium network. Furthermore, we explored the gut viral signatures for disease discrimination and achieved an area under the receiver operator characteristic curve (AUC) of above 0.95, suggesting the potential of the gut virome in the prediction of SLE.

**Conclusion:**

Our findings demonstrated the alterations in viral diversity and taxonomic composition of the gut virome of SLE patients. Further research into the etiology of SLE and the gut viral community will open up new avenues for treating and preventing SLE and other autoimmune diseases.

## Introduction

Systemic lupus erythematosus (SLE) is a prototypical autoimmune disease. The disease is characterized by chronic inflammation, which leads to tissue and organ damage, and is more common in females (1). The etiology of SLE is unknown, but it is influenced by several confounding factors, including genetic, environmental, and epigenetic factors (2, 3). These factors can cause immune system abnormalities, including abnormal B cells that produce autoantibodies, autoreactive T cells, and pro-inflammatory cytokines (4). A single factor cannot cause SLE, as a combination of microbial communities and environmental stress is also associated with SLE (5, 6). A recent metagenomic study found that imbalances in the microbial community may be associated with the development of SLE (7). Hevia et al. described the imbalance of gut microbiota in SLE patients (8), and Ma et al. proved that the fecal microbiota of SLE mice could stimulate the inflammatory response and change the expression of SLE susceptibility genes through fecal bacteria transplantation experiment (5). These studies suggest that gut microbiota is associated with SLE and may even be one of the inducing factors of SLE. Since the gut microbiota contains a variety of components (e.g., bacteria, viruses, and fungi), an obvious blind spot in the above-mentioned studies is what function each component plays.

Despite the fact that current research focuses on gut bacteria as one of the main components, gut viruses are also noteworthy. The human gut virome is mainly composed of bacteriophages, especially the *Caudovirales* order (9). Bacteriophages infect bacteria, co-evolve with the gut microbiome, and play an important role in shaping the composition of the gut microbiome. They aid nutrient turnover and facilitate horizontal gene transfer in the gut microbiome (10, 11). Virome may have alterations in response to changes in human gut microbiome composition in many diseases closely related to the gut microbiome. Recent studies have highlighted the association of gut virome with many diseases, particularly diabetes (12), inflammatory bowel disease (IBD) (13), and autoimmune diseases (14). In addition, gut virome can also directly affect the body’s health through the immune system (15, 16). Thus, understanding the changes in gut virome diversity and structure during disease is crucial to exploring the potential pathogenesis of the disease.

However, due to metagenomic sequencing technology, some viruses with low abundance or difficult sequencing are easily overlooked. Therefore, gut virome studies will also use virus-like particle (VLP) metagenomic sequencing (17–19). The integration of whole metagenomic and VLP metagenomic sequencing analysis can obtain a more comprehensive map of the gut virus population. To identify changes in the gut virome in SLE, we collected fecal samples and analyzed the viral communities from 16 SLE patients and 31 healthy subjects. Specifically, we compared the viral composition of SLE patients and healthy individuals based on both bulk metagenomic and VLP-based sequencing data, and the relationships between viruses and bacteria were also investigated. A better understanding of the etiology and pathogenesis of SLE based on gut virome exploration will contribute to the development of new prevention and treatment strategies.

## Methods

### Ethics statement, subject recruitment, and sample collection

The protocol of this study was approved by the Medical Ethics Committees of the Second Affiliated Hospital of Guizhou University of Traditional Chinese Medicine. All subjects who took part in this study gave written informed consent. This study enrolled sixteen SLE patients from the Department of Rheumatology and Immunology, the Second Affiliated Hospital of Guizhou University of Traditional Chinese Medicine, from August 2020 to August 2021. The demographic and clinical characteristics of the SLE patients are shown in **Table S1**. All patients fulfilled the 2019 European League Against Rheumatism/American College of Rheumatology (EULAR/ACR) classification criteria for SLE (20). The exclusion criteria were as follows: (1) SLE patients with neuropsychiatric lupus, renal lupus, vasculitis, pancreatitis, enteritis, myocarditis, or severe major systemic diseases such as malignancy, pyemia, and cardiovascular or metabolic disorders; (2) patients with overlap syndromes; (3) patients with diarrhea; (4) patients who received antibiotics or probiotics treatment in 1 month; (5) patients who had drunk sour milk within 1 week or had smoking or drinking habits. Thirty-one age-matched and gender-matched healthy subjects were recruited based on records available from the Department of Medical Examination Center, Second Affiliated Hospital of Guizhou University of Chinese Medicine. All volunteers’ feces were immediately transported to the laboratory on dry ice and preserved in two aliquots at -80°C until analysis.

### Virus-like particles enrichment and virome DNA extraction

Virus-like particles were enriched from the fecal samples according to our previous methods (21). Briefly, for each sample, 0.1 g feces were added into 1 ml HBSS buffer (137 mM NaCl, 5.4mM KCl, 1.3 mM CaCl_2_, 0.3 mM Na_2_HPO_4_·2H_2_O, 0.5 mM MgCl_2_·7H_2_O, 0.4 mM KH_2_PO_4_, 0.6 mM MgSO_4_·7H_2_O, 4.2 mM NaHCO_3_, 5.6 mM D-glucose). To remove debris and bacterial cells from fecal suspensions, samples were centrifuged twice at 10,000 g for 2 minutes at 8°C and the supernatants were then passed through 0.45 μm and 0.22 μm filters successively. The sterile filtrate was mixed with an equivalent amount of HBSS buffer and centrifuged for one hour at 750,000 g (Sorvall mTX150, Thermo Scientific) for 1 hour. The remaining, non-encapsulated nucleic acid in centrifugal precipitation was degraded by treating with a mixture of 2.4 μl TURBO DNase (4.8 U, Invitrogen), 8 μl RNase A/T1 Mix (16 μg RNase A, 40 U RNase T1, Thermo Scientific), and 1 μl Benzonase (5 U, EMD Millipore), followed by heat inactivation of nucleases at 65°C for 10 min.

The TIANamp Virus DNA/RNA Kit (TIANGEN) was used to extract viral DNA from the enriched virus-like particles according to the manufacturer’s protocols. A mixture with 11.5 μl DEPC H_2_O, 1 μl 20mM random primers D2-8N(5’-AAGCTAAGACGGCGGTTCGGNNNNNNNN-3’), 1 μl 10xRT mix, 1 μl 10mM dNTP, and 11.5 μl extracted viral DNA was prepared to synthesize the first strand of viral DNA. Then, we denatured the enzyme of the mixture at 95 °C for 5 minutes and added the Klenow fragment solution (0.15 μl 10x Klenow Buffer, 0.5 μl Klenow fragment, 0.85μl DEPC H2O) at 37 °C. To obtain second-strand viral DNA, the procedure was repeated twice.

### Shotgun sequencing of viromes and bulk metagenomes

For bulk metagenomes, total metagenomic DNA was extracted from approximately 170 mg of feces using standard methods (22). For each bulk or virome DNA sample, we prepared a library using the NEB Next^®^ Ultra™ DNA Library Prep Kit for Illumina (NEB, USA). Briefly, the fresh genomic DNA samples were mechanically fragmented by sonication to a size of approximately 350 bp. After end-polished, A-tailed, and ligated with the full-length adapter, the DNA fragments were then amplified using PCR. The AMPure XP system (Beckman Coulter, Beverly, USA) was used to purify the PCR products. After that, the DNA libraries were shotgun sequenced based on the Illumina NovaSeq platform, which generated raw 2 × 150 bp paired-end reads for further analysis. Initial base calling of the metagenomic dataset was processed using the sequencing platform’s system default parameters. The raw paired-end reads for each sample were independently processed for quality control using fastp (23). Fastp processed with the raw reads by trimming the low-quality (Q<30) bases at the end of reads and filtering ‘N’-containing (>3 ‘N’ bases), adapter-contaminated or short length (<90 bp) reads, to generate the high-quality reads. The human reads were removed from the high-quality reads set based on their alignment to the human reference genome (GRCh38) using Bowtie2 (24).

### Identification and clustering of viral sequences

High-quality clean reads for each bulk or virome metagenomic sample were performed for *de novo* assembly *via* MEGAHIT (25) with a broad range of k-mer sizes (–k-list 21,41,61,81,101,121,141). For bulk metagenome samples, all assembled contigs with a length ≥2 kbp were firstly assessed by using CheckV (26), and the non-viral contigs were removed if their viral gene count was less than the number of microbial genes. Then, we identified potential viral sequences from the remaining contigs based on two criteria: 1) contigs with *P*-value <0.01 and score >0.90 in DeepVirFinder (27); and 2) contigs identified as viruses by VIBRANT (28) with default options (-meta mode). Low-quality or “not-determined” viral contigs assessed by CheckV were further removed to avoid contamination. For virome metagenome samples, we identified potential viral sequences from the assembled contigs with length ≥2 kbp based on the following criteria: 1) contig whose viral gene was more than the number of microbial genes in CheckV (26); 2) contig with *P*-value <0.01 and score >0.90 in DeepVirFinder (27); and 3) contig identified as a virus by VIBRANT (28) with default options (-virome mode). According to the previous study (29), we searched for bacterial universal single-copy orthologs (BUSCO) (30) within viral sequences using hmmsearch (31) with the default options and calculated the ratio of the number of BUSCO to the total number of genes in each viral sequence (referred to as the BUSCO ratio). After removing highly contaminated viral sequences with a ≥5% BUSCO ratio, the remaining viral sequences were considered the final viral sequences for each sample.

The viral sequences were de-replicated using the following procedures: 1) all viral sequences were aligned pairwise using BLASTN with the options ‘-evalue 1e-10 -word size 20 -num alignments 99999’; 2) viral sequences that shared 95% nucleotide identity across 75% of the sequence were clustered into a viral operational taxonomic unit (vOTU) using in-house scripts.

### Taxonomy assignment and host prediction of viruses

Viral protein-coding genes were called from the viral sequences using Prodigal (32). Taxonomic annotation of viral sequences was carried out based on protein sequence alignment to the combined database derived from the Virus-Host DB (downloaded in May 2021) (33), *crAss-like* protein sequences from Guerin’s study (34), and viral protein sequences from Benler’s study (35). To implement accurate family-level taxonomy, we first aligned proteins of viral sequences from NCBI RefSeq against the combined database using DIAMOND (36) with the parameters ‘–query-cover 50 –subject-cover 50 –id 30 –min-score 50 –max-target-seqs 10’. A viral sequence was annotated to the viral family level when over a quarter of its proteins were matched to the same family.

The virus-host prediction was performed using two bioinformatic methods that included prophage prediction and clustered regularly interspaced short palindromic repeats (CRISPR)-spacer matches. For prophage prediction, the viral sequence was blasted against the gut prokaryotic genes from the comprehensive unified human gastrointestinal genome (UHGG) database (37), and a host was assigned if the viral sequence matched the host genome at 90% nucleotide identity and 30% viral coverage (29). For CRISPR-spacer matches, we first predicted CRISPR spacer sequences from the UHGG genomes using MinCED (38) with the option ‘-minNR 2’, and then assigned a host to the virus if the host CRISPR spacer sequence was matched to the viral genome (bit-score ≥45) using BLASTN with options ‘-evalue 1e-5 -word_size 8 -num_alignments 99999’ (29).

### Analyses of the gut bacteriome

The bacteriome composition of each fecal sample was profiled based on the UHGG database (37), which comprised 204,938 nonredundant genomes from 4,644 gut prokaryotes. The bulk metagenomic reads for samples were aligned against the UHGG genomes to generate the gut bacteriome profiles. Relative abundances of 4,644 prokaryotic species were calculated by normalizing for each sample, and the relative abundances at the phylum and genera levels were obtained by summing the abundances of species from the same taxa.

### Statistical analyses

Statistical analyses were implemented on the R platform (https://www.r-project.org/). The Shannon diversity index for the gut viral composition was calculated based on the relative abundance profile at the species level using the *vegan* package in the R platform. The *adonis* function of the *vegan* package was used to conduct the permutational multivariate analysis of variance (PERMANOVA), and the *adonis P*-value was calculated based on 1,000 permutations. Effect size analysis of the microbiome and virome profiles was performed following the previous study (39). Distance-based redundancy analysis (dbRDA) was performed on normalized taxa abundance matrices with R *vegan* package according to Bray-Curtis distances. Random forest models were trained using the *randomForest* package (1,000 trees) to distinguish between SLE patients and healthy controls. The performance of the predictive model was evaluated using receiver operator characteristic (ROC) analysis, which was implemented on the R platform with the *pROC* package. The area under the receiver operator characteristic curve (AUC) was calculated from the ROC analysis. For multiple testing, *P*-values were adjusted to obtain the false discovery rate (FDR) using the Benjamini-Hochberg procedure.

Correlation analysis of the gut viruses and bacteria was performed based on Spearman’s rank correlation coefficient. A correlation coefficient was calculated based on Spearman correlation coefficient *ρ* > 0.6 or < -0.6 and correlation test adjusted *P* < 0.05. The correlation network was visualized using Cytoscape (40).

## Results

### Overview of the gut virome in SLE patients and healthy subjects

**Figure 1:**
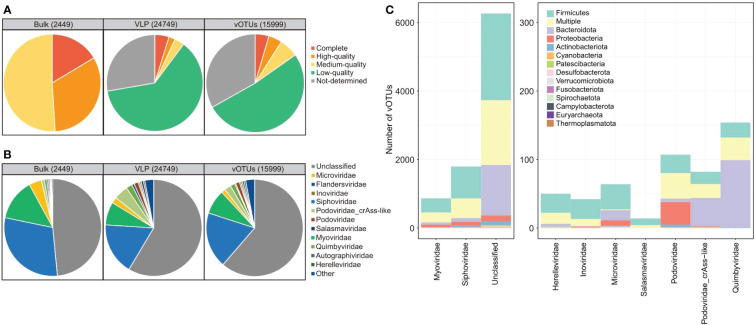
Summary of quality assessment, taxonomic assignment, and host prediction of viral sequences. **(A, B)** Pie plot showing the quality assessment **(A)** and taxonomic assignment **(B)** of the viral sequences identified from bulk and virome metagenomic datasets and the vOTUs. **(C)** The distribution of prokaryotic hosts of all vOTUs generated in this study. The vOTUs are organized by their family-level taxonomic assignment, and the host taxa are displayed by phylum. The number of vOTUs with multiple predicted hosts is indicated in yellow.

To characterize the gut virome in patients with SLE, we performed bulk metagenomic sequencing and VLP-based viral metagenomic sequencing on fecal samples from 16 SLE patients and 31 healthy controls. Bulk metagenomic sequencing obtained 429.2 Gbp of high-quality data (9.1 ± 2.7 Gbp per sample; **Table S2**). A total of 780,695 contigs (length ≥ 2 kbp) were generated after *de novo* assembly for each sample, and 2,449 of these contigs were recognized as highly credible viral sequences based on their sequence features and homology to known viral genomes (see Methods). The average length of the contigs was 36,669 bp, with a minimum and maximum length of 2,126 and 346,124 bp, respectively. 16.4% of viral sequences were assessed as complete viral genomes based on the CheckV algorithm (26), and the remaining 32.6% and 51.0% of sequences were assessed as high-quality (completeness > 90%) and medium-quality (completeness > 50%) viruses, respectively (**Figure 1A**).

VLP-based viral metagenomic sequencing obtained 219.1 Gbp of high-quality non-human data (4.7 ± 1.8 Gbp per sample; **Table S2**). The dataset was assembled into 97,797 contigs (length ≥ 2 kbp), and 24,749 of these contigs were recognized as highly credible viral sequences. The average length of the contigs was 6,889 bp (N50 length, 10,404 bp), with a minimum and maximum length of 2,000 and 262,173 bp, respectively. Only 10.1% of viral contigs were assessed as complete (4.8%), high-quality (2.1%), or medium-quality (3.2%) viral genomes based on the CheckV algorithm (26), and the remaining 89.9% of viral sequences were low-quality or undetermined viruses (**Figure 1A**).

The viral contigs from both bulk and viral metagenomes were then grouped into 15,999 vOTUs by clustering the contigs at 95% nucleotide similarity. These vOTUs included 4.5% complete viral genomes, 4.4% high-quality viral genomes, 6.3% medium-quality viral genomes, and 84.8% low-quality or undetermined viruses (**Figure 1A**). 38.2% (6,105/15,999) of all vOTUs could be taxonomically assigned to a viral family. *Siphoviridae*, *Myoviridae*, *Microviridae*, *Quimbyviridae*, and *Podoviridae* were the dominant families for these vOTUs (**Figure 1B**). And the finding was consistent in the viral sequences from both bulk and viral metagenomic datasets. Furthermore, we predicted the microbial hosts for the vOTUs and found that 60.2% (9,636/15,999) of vOTUs could be assigned to one or more host bacteria or archaea. The hosts of these viruses were mostly members of Firmicutes, Bacteroidota, Proteobacteria, and Actinobacteriota (**Figure 1C**).

### Comparison of the viromes between patients and controls in the bulk metagenomic dataset

**Figure 2:**
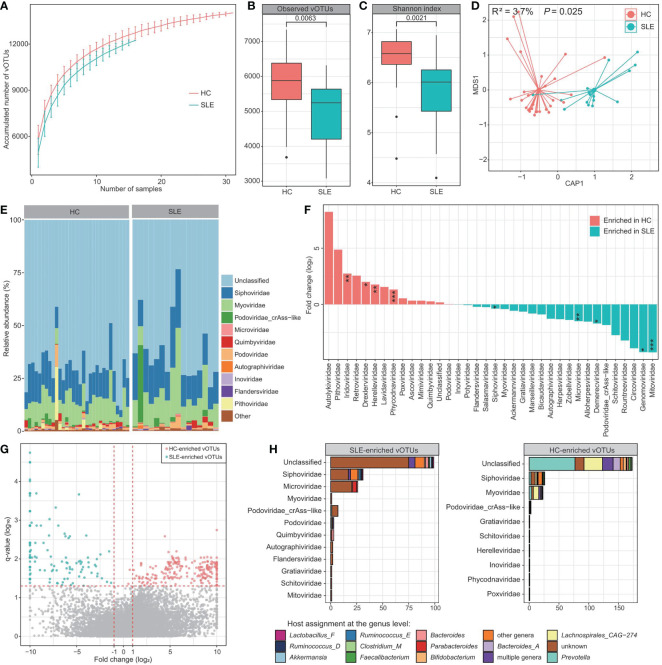
Difference in bulk gut virome between SLE patients and healthy controls. **(A)** Rarefaction curve analysis of the number of vOTUs on each group of samples. The number of identified vOTUs in different groups is calculated based on a specific sample size with 10 random replacements, and the median and quartile numbers are plotted. **(B, C)** Boxplot showing the observed number of vOTUs **(B)** and Shannon diversity index **(C)** between two groups. The significance level is calculated using the Student’s *t* test. **(D)** dbRDA analysis based on the gut viral composition at the vOTU level, revealing the separations between SLE patients and healthy controls. The location of samples (represented by filled nodes) in the first two multidimensional scales are shown. Lines connect samples that belonged to the same group. **(E)** Composition of bulk gut virome at the family level. **(F)** Barplot showing the fold changes of viral families when compared between patients and controls. The significance level in the Wilcoxon rank-sum test is denoted as: * adjusted *P* < 0.05, ** adjusted *P* < 0.01, *** adjusted *P* < 0.001. **(G)** Volcano plots showing the fold change vs. q-values for all vOTUs when compared between patients and controls. The X-axis shows the ratio (log2 transformed) of vOTU abundance in SLE patients (fold < 0) compared with that in healthy controls (fold > 0). The Y-axis shows the q-value (-log10 transformed) of a vOTU. Horizontal dotted lines: adjusted *P* < 0.05; vertical dotted lines: fold < -2 and fold > 2. **(H)** Taxonomic and host assignment of the SLE-enriched (left panel) and control-enriched (right panel) vOTUs. The vOTUs are grouped at the family level, and their hosts are shown at the genus level.

To explore signatures of the gut virome in SLE, first, we compared the viral profiles between the SLE patients and healthy controls based on the bulk metagenomic dataset. Rarefaction analysis showed that, at the same sample size, the number of vOTUs observed in SLE patients was significantly lower than that in healthy controls (**Figure 2A**). Consistently, within-sample alpha diversity analysis revealed that both the viral richness (estimated by the observed number of vOTUs) and diversity (estimated by the Shannon index) had significantly decreased in SLE patients compared with controls (**Figures 2B, C**). Next, dbRDA analysis based on the Bray-Curtis distance at the vOTUs level revealed a clear separation between the two groups (**Figure 2D**), with the disease state explained an effect size of 3.7% (PERMANOVA *p*=0.025) on the gut virome variations.

Most of the gut virome of both SLE patients and healthy controls could not be assigned to known viral families (average relative abundance, 65.2% ± 4.5%; **Figure 2E**). Apart from this, *Siphoviridae* (11.1% ± 1.0%), *Myoviridae* (7.3% ± 0.8%), *crAss-like* [5.7% ± 1.8%; a *Podoviridae* subclade with unique genomic and functional features (41, 42)], and *Microviridae* (3.6% ± 1.2%) were the most dominant families in the viromes of all investigated samples, followed by a small proportion of *Podoviridae*, *Quimbyviridae*, *Autographiviridae*, and *Inoviridae* (**Figure 2E**). Using the Wilcoxon rank-sum test, we identified 9 families with significant differences in their relative abundances between the patients and controls (adjusted *P* < 0.05). 4 of these families, including *Iridoviridae*, *Drexlerviridae*, *Herelleviridae*, and *Phycodnaviridae*, were significantly enriched in the virome of SLE patients, whereas 5 families, including *Siphoviridae*, *Mitoviridae*, *Genomoviridae*, *Demerecviridae*, and *Microviridae*, were enriched in the virome of healthy controls (**Figure 2F**). At the vOTUs level, 408 vOTUs were identified with significant differences in their relative abundances between patients and controls (Wilcoxon rank-sum test adjusted *P* < 0.05). Among these, 177 vOTUs were significantly enriched in SLE patients, and 231 vOTUs were depleted (**Figure 2G**; **Table S3**). The SLE-enriched vOTUs included 31 members of *Siphoviridae*, 26 *Microviridae*, 7 *crAss-like*, 3 *Podoviridae*, 3 *Quimbyviridae*, and 120 unclassified viruses, while the control-enriched vOTUs were composed of 26 *Siphoviridae*, 23 *Myoviridae*, and 173 unclassified viruses (**Figure 2H**). SLE-enriched vOTUs were frequently assigned to phages of *Bacteroides*, *Bacteroides_A*, *Parabacteroides*, and *Ruminococcus_E*, while the control-enriched vOTUs had remarkably higher proportions of *Prevotella* and *Lachnospirales_CAG-274* phages.

### Comparison of the viromes between patients and controls in VLP-based viral metagenomic dataset

**Figure 3:**
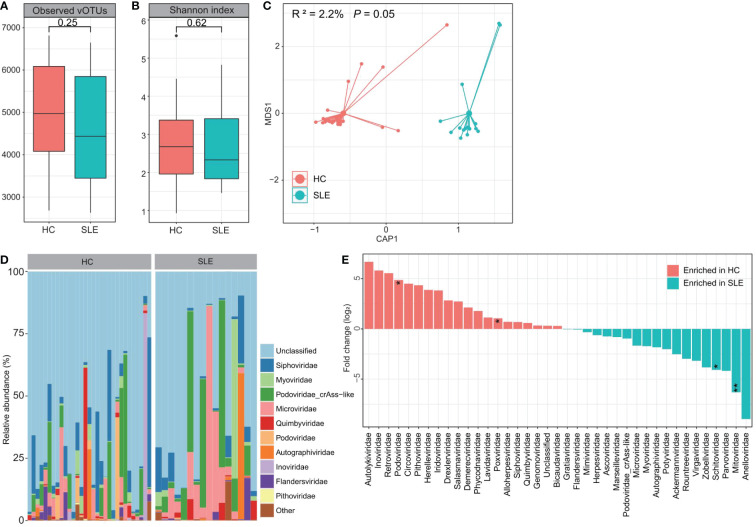
Difference of VLP-based gut virome between SLE patients and healthy controls. **(A, B)** Boxplot showing the observed number of vOTUs **(A)** and Shannon diversity index **(B)** between two groups. The significance level is calculated using the Student’s *t* test. **(C)** dbRDA analysis based on the gut viral composition at the vOTU level reveals the separations between SLE patients and healthy controls. The location of samples (represented by filled nodes) in the first two multidimensional scales are shown. Lines connect samples that belonged to the same group. **(D)** Composition of VLP-based gut virome at the family level. **(E)** Barplot showing the fold changes of viral families when compared between patients and controls. The significance level in the Wilcoxon rank-sum test is denoted as: * adjusted *P* < 0.05, ** adjusted *P* < 0.01.

Next, we compared the gut viromes between the SLE patients and healthy controls based on the VLP metagenomic dataset to investigate the signatures of free viral particles. In VLP metagenomic samples, there was no significant difference in the observed number of vOTUs (viral richness) or Shannon diversity index between SLE patients and healthy subjects (**Figures 3A, B**). However, similar to the bulk metagenomes, dbRDA analysis based on the Bray-Curtis distance of viral metagenomes at the vOTU level also revealed visible separation between the two groups (effect size = 2.2%, PERMANOVA *P* = 0.05; **Figure 3C**), suggesting a considerable gut viral dysbiosis in these SLE patients.

At the family level, a large proportion of the virome in both groups was assigned to unclassified viruses (average relative abundance = 62.8% ± 7.4%; **Figure 3D**). *crAss-like* (9.8% ± 3.0%), *Siphoviridae* (7.6% ± 1.4%), *Microviridae* (7.0% ± 2.1%), *Myoviridae* (2.6% ± 0.9%), *Quimbyviridae* (2.3% ± 0.8%), and *Autographiviridae* (2.0% ± 0.9%) were the most dominant families in the remaining viral communities of all investigated samples. The Wilcoxon rank-sum test showed that *Mitoviridae* and *Schitoviridae* were significantly enriched in the gut virome of SLE patients compared with that of controls, while *Podoviridae* and *Poxviridae* were enriched in healthy controls (**Figure 3E**). At the vOTU level, we observed significant differences in the abundances of 18 vOTUs between the SEL patients and healthy controls (Wilcoxon rank-sum test adjusted *P* < 0.05; **Table S4**). Seventeen of these vOTUs, including 3 members of the *Siphoviridae*, 1 *Autographiviridae*, 1 *Flandersviridae*, 1 *Myoviridae*, 1 *Podoviridae*, and 10 unclassified viruses, were enriched in patients, and only one unclassified virus was enriched in the control group.

### Correlation analysis of gut viruses and bacteria

**Figure 4:**
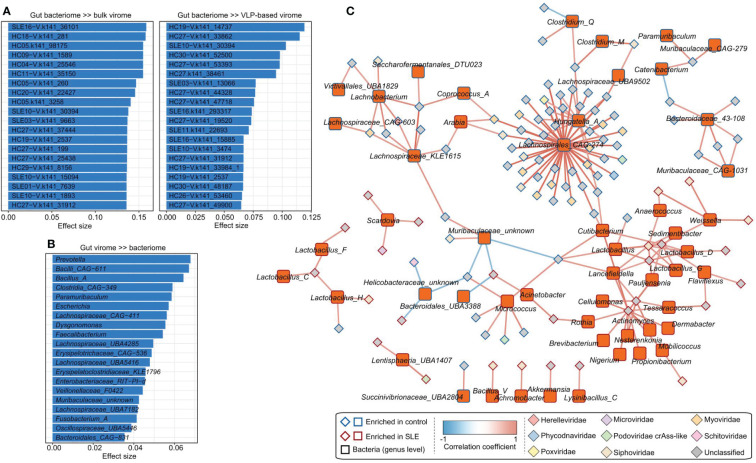
Associations between gut virome and bacterial microbiome. **(A)** The vOTUs with the highest effect size are significantly impacted by the gut bacteriome. The top 20 vOTUs from the bulk metagenomic dataset (left panel) and the top 20 vOTUs from the VLP-based virome dataset (right panel) are shown. **(B)** The top 20 bacterial genera for which the highest effect size has a significant impact on the gut virome. **(C)** The correlation network between SLE-associated vOTUs and bacterial genera. The network was constructed based on Spearman correlations (*ρ* > 0.6 or < -0.6, correlation test adjusted *P* < 0.05) between viruses and bacteria.

To study the virus-bacterium correlations, first, we performed a PERMANOVA-based analysis to estimate the effect size of variances between the gut virome and bacteriome. The analysis showed that the gut bacteriome accounted for a considerable proportion of the variance of many vOTUs in both the bulk and VLP-based virome datasets. For example, the bacteriome explained over 15% of the variances for 6 vOTUs in the bulk virome and over 10% of the variances for 3 vOTUs in the VLP-based virome (**Figure 4A**). On the other hand, several bacterial genera, including *Prevotella*, *CAG-611*, and *Bacillus_A*, had the largest effects on the gut virome (**Figure 4B**).

Next, we calculated the correlation coefficients between 426 SLE-associated vOTUs (408 identified from bulk metagenomes and 18 from VLP-based viral metagenomes) and 841 bacterial genera. This analysis identified a total of 189 significant correlations between 106 vOTUs and 56 bacterial genera (**Figure 4C**). A large proportion of vOTUs was positively correlated with *Lachnospirales_CAG-274* and *Hungatella_A*, suggesting their key roles in the virus-bacterium network.

### Random forest model predicts SLE state

**Figure 5:**
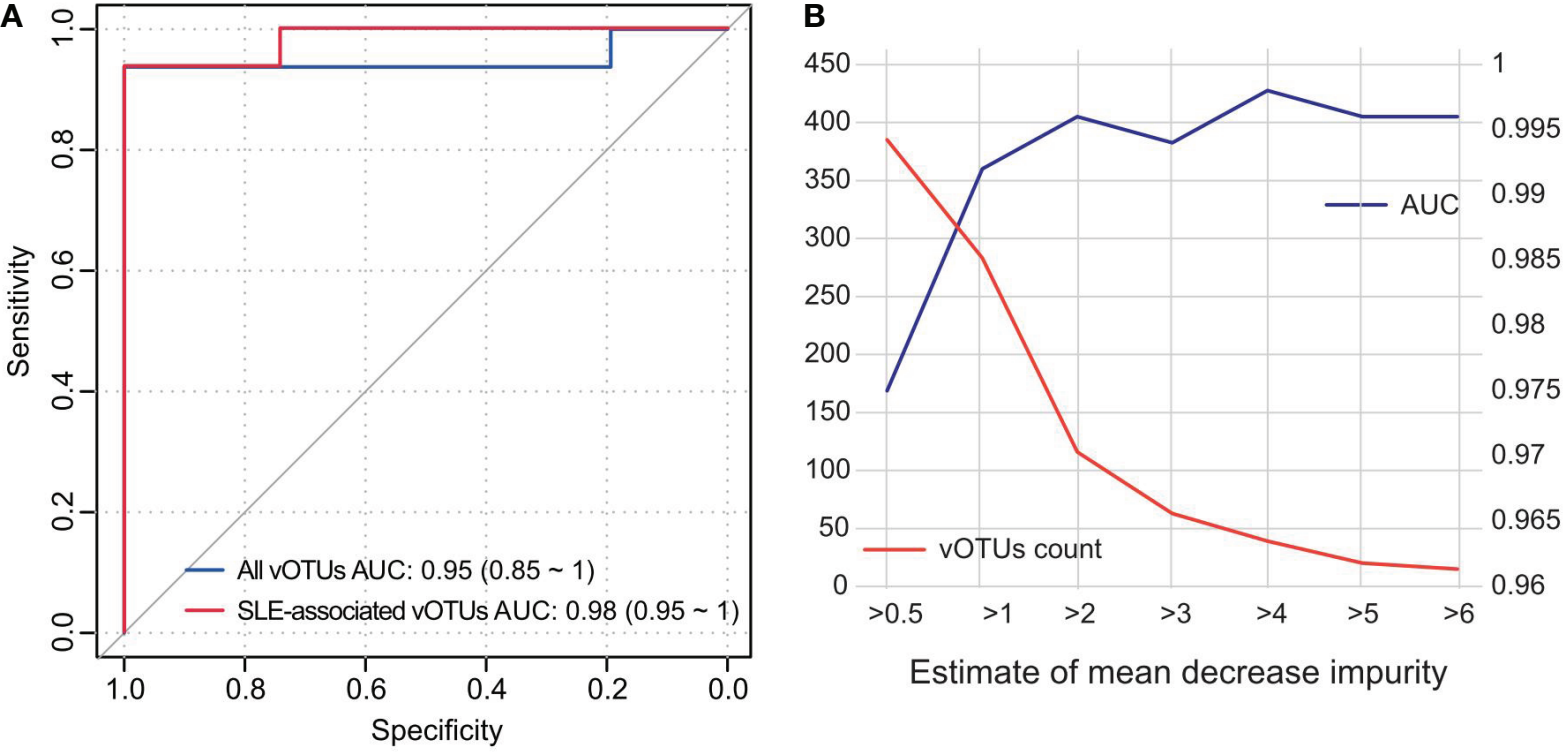
Classification of SLE status by the abundance of gut virome. **(A)** ROC analysis for the classification of SLE patients and healthy controls. The performances of models trained and tested by all vOTUs and 426 SLE-associated vOTUs are shown. **(B)** AUC values for different numbers of vOTUs used in the random forest models. The number of vOTUs is shown by the red line on the left axis, and the AUCs are shown by the blue line on the right axis.

To assess the discriminating effect of the gut virome on SLE status, we used all vOTUs as predictors of the random forest model. The model obtained an AUC of 0.95 (95% confidence interval [CI], 0.85-1.00; **Figure 5A**) in distinguishing healthy patients from SLE patients. Retraining the model using 426 SLE-associated vOTUs also showed that the prediction effect is slightly improved with an AUC of 0.98 (95% CI, 0.95-1.00). Moreover, to minimize the number of viruses used in the model, we selected a subset of vOTUs to train the random forest model based on the importance of all vOTUs. This analysis showed that the model obtained the highest AUC (0.998) when using a subset of 39 top important vOTUs (**Figure 5B**). These findings suggest the high diagnostic potential of the gut virome in SLE discrimination.

## Discussion

In this study, we revealed the changes in gut viral populations in 16 SLE patients compared with 31 healthy controls. Our study strengthened the previous bulk metagenome-based study on the gut virome of autoimmune diseases (including SLE) (14) by adding more information from both bulk and VLP metagenomic sequencing technologies. VLP-based virome sequencing has improved the diversity of viruses observed, allowing information on previously overlooked viruses to be captured (21).

In the bulk metagenomic dataset, we identified 4 viral families, including *Iridoviridae*, *Drexlerviridae*, *Herelleviridae*, and *Phycodnaviridae*, that were significantly enriched in the SLE virome, whereas 5 families, including *Siphoviridae*, *Mitoviridae*, *Genomoviridae*, *Demerecviridae*, and *Microviridae* were enriched in healthy controls. *Iridoviridae*, a family of large and icosahedral viruses that are known to infect ectothermic vertebrates such as bony fish, amphibians, and reptiles (43), have frequently been found in human fecal or blood samples (44–47) and reported as one of the most abundant viral families in the gut of human immunodeficiency virus (HIV) infected patients (45). Viral phages belonging to the *Drexlerviridae* family, such as KM18 and IME268, have cleavage activity against *Klebsiella pneumoniae* due to their putative endosialidase (depolymerase) enzyme activity (48, 49), and more relevantly, *Klebsiella* was reported significantly enriched in SLE patients from China (50). *Herelleviridae* is a bacterial virus that infects members of the Firmicutes, especially the *Enterococcus* genus (51). *Phycodnaviridae* is a large icosahedral dsDNA viral family that can infect algae (52), and it has also been found in the oropharyngeal samples obtained from healthy adults (53). The enrichment of these families in SLE patients suggested that they may play potential roles in subjects with an immunocompromised state.

By comparing the viromes between patients and controls in the VLP-based viral metagenomic dataset, we found that *Mitoviridae* and *Schitoviridae* were significantly enriched in the gut virome of SLE patients compared with that of controls, while *Podoviridae* and *Poxviridae* were enriched in healthy controls. *Schitoviridae* was a new family of N4-like phages, and there was a poorly reported association between *Schitoviridae* and human disease (54). Analysis of phages and autoimmune disease (specifically the patients with RA and SLE) in 476 Japanese showed that *Podoviridae* were significantly decreased in the gut of the patients with SLE, and it has a symbiotic relationship with *Faecalibacterium* (14). These results were consistent with our studies.

In the viral host aspect, we found the viruses that are predicted to infect *Bacteroides*, *Bacteroides_A*, *Parabacteroides*, and *Ruminococcus_E* were enriched in the SLE patients. Consistently, Chen et al. found that *Bacteroides fragilis* was enriched in the SLE gut microbiota and reduced after treatment (7). In a cross-sectional cohort study, Azzouz et al. found that the relative abundance of *Ruminococcus gnavus* in patients with higher SLE disease activity index was overall 5-fold greater than that in healthy subjects (55). These findings suggest that the abundance of bacteria may also affect phages by providing their hosts.

Notably, different research strategies based on bulk and VLP metagenomic datasets have their pros and cons. The enriched VLPs could improve the sequencing depth for viruses, while the bulk metagenome is easier to preprocess and contains bacterial information, which is important for the analysis of the relationship between bacteria and viruses. Until now, many attempts to screen SLE biomarkers have been made. Based on bacterial 16S rRNA gene (56) and various immune cells (57), 0.79 and 0.96 of AUCs were achieved, respectively. We obtained a more accurate and higher AUC of 0.98 (**Figure 5**) using only 426 gut vOTUs. This analysis showed that the model obtained the highest AUC (0.998) when using a subset of 39 top important vOTUs. These findings are encouraging developments that suggest the high diagnostic potential of the gut virome in SLE discrimination.

A major limitation of this study was the small sample size (16 SLE patients vs. 31 healthy controls). Future studies with a larger cohort of patients and controls will be needed to further uncover the relationship between the gut viral community and SLE. On the other hand, due to the lack of comprehensive reference databases, the majority of viruses in the human gut remain unknown. Many unclassified viruses will be taxonomically classified into known or newly-identified taxa, which will result in more accurate descriptions of the virome characterization of SLE patients.

## Conclusion

Overall, based on bulk and VLP-based shotgun metagenomic sequencing datasets, our results systematically characterized the gut virome in SLE patients. Some viral signatures had significantly different abundances between the SLE patients and healthy subjects. Importantly, the excellent predictive model (AUC >0.95) using only a small number of viruses heralded its potential for clinical application. Our research will bring the revelation for future mechanistic and clinical intervention studies.

## Data availability statement

The datasets presented in this study can be found in online repositories. The names of the repository/repositories and accession number(s) can be found below: https://www.ebi.ac.uk/ena, PRJEB55711.

## Ethics statement

The studies involving human participants were reviewed and approved by the Medical Ethics Committees of the Second Affiliated Hospital of Guizhou University of Traditional Chinese Medicine. The patients/participants provided their written informed consent to participate in this study.

## Author contributions

CC, QY, XY, WS and WM contributed to conception and design of the study. CC, QY and SL drafted the manuscript. QZho, FT, ZL, YH, CL, DZ and HL collected the samples and information. SL, QL, GW, AZ, HU, YZ, YA, JZ and QZha performed the data analysis and investigation. All authors revised the manuscript, contributed to the article, and approved the submitted version.
